# India can consider integration of three eliminable disease control programmes on malaria, lymphatic filariasis, and visceral leishmaniasis

**DOI:** 10.1371/journal.ppat.1009492

**Published:** 2021-05-20

**Authors:** Manju Rahi, Rini Chaturvedi, Payal Das, Amit Sharma

**Affiliations:** 1 Indian Council of Medical Research, New Delhi, India; 2 International Centre for Genetic Engineering and Biotechnology, New Delhi, India; 3 ICMR-National Institute of Malaria Research, New Delhi, India; University at Buffalo School of Medicine and Biomedical Sciences, UNITED STATES

## Introduction

Disease-specific programmes (also called vertical, stand-alone, categorical, or free-standing) are directed, supervised and executed via single vehicle using dedicated health workers. In contrast, integrated programmes (also known as horizontal) aim to tackle the overall health problems on a wider front and on a longer-term basis through the creation of permanent multifunctional healthcare delivery institutions [1]. Several disease control programmes like the Global Fund to Fight AIDS, Tuberculosis and Malaria (GFTAM), Roll Back Malaria (RBM), Global Polio Eradication Initiative (GPEI) and Expanded Programme of Immunization (EPI) are vertical programmes focused on malaria, polio and other preventable diseases respectively [1]. GFTAM and other organizations have been referred to as agencies with their parallel systems of reporting, monitoring and so on. However, there are inherent differences among these agencies. Global Fund is an international organization which in partnership with national governments (including India), civil society, technical agencies, private sector and others invests significantly in scaling up preventive and management tools of TB, malaria and AIDS. Whereas other organizations like CARE India and KalaCORE India have joined hands with the government to support implementation of certain components of kala azar research and elimination programme in some endemic states of India. Vertical programmes for diseases like malaria, tuberculosis, leprosy, filaria, trachoma and cholera have been existent in India since long [2]. However, India still suffers from a significant burden of 3 parasitic vector-borne diseases, namely malaria, visceral leishmaniasis and lymphatic filariasis. Malaria is prevalent in a total of 747 districts* with approximately 698 million population at risk [3]. Visceral leishmaniasis, also known as kala azar (black fever), is endemic in 54 districts of Bihar, Jharkhand, Uttar Pradesh and West Bengal putting 130 million population at risk [4]. Lymphatic filariasis is prevalent in 256 districts and affects >23 million people with nearly 650 million people at risk of acquiring the infection in India [5]. Currently, malaria, visceral leishmaniasis and lymphatic filariasis are targeted for elimination (visceral leishmaniasis and lymphatic filariasis by 2021 and malaria by 2030) [6]. Elimination programmes pertaining to these diseases are important in the health system of India and consume substantial resources.

The National Vector Borne Disease Control Programme (NVBDCP) is a focal agency of the Government of India responsible for control of 6 vector-borne diseases, i.e., malaria, visceral leishmaniasis, lymphatic filariasis, dengue, chikungunya and Japanese encephalitis. NVBDCP is a vertical programme due to separate budgets, healthcare cadres, supply chains, health information systems, monitoring, and evaluation frameworks but partially embedded in the existing mainstream healthcare system of the country at the grassroot level, especially after the advent of National Rural Health Mission (NRHM).

The verticality of the 3 programmes within NVBDCP is evident in terms of staff, infrastructure and operations. Despite some attempts at integration at district level, State Programme Officers (SPOs) are different for each disease, and separate infrastructures like Filaria units still exist in some states. Vector control activities like indoor residual spray (IRS) (common for malaria and visceral leishmaniasis) and the use of larvicides (common for lymphatic filariasis and malaria) are done in an independent and disconnected fashion. The grassroots-level workers and Primary Health Centre (PHC) staff are the same for surveillance of all three, but the training programmes are segregated for each disease which does now allow practice of joint surveillance. Finally, the key aspect of surveillance in terms of data (reporting formats and health information management systems) are different and not shared. NVBDCP provides antimalarial drugs, insecticides and larvicides to the states. Operational costs of the implementation of the programme are borne by states. The implementation of the national programme is thus the responsibility of states. Similarly, for kala azar, the central government provides drugs, insecticides and technical support and the states bear implementation costs. Lymphatic filariasis programme is a centrally assisted programme including procurements of drugs for MDA and other operations.

Despite the above verticality, the above programmes utilize the same network of healthcare delivery system in common areas of endemicity. The National Rural Health Mission was launched in 2005 (which was later subsumed in National Health Mission in 2013) and aimed at providing decentralised community-owned healthcare delivery system through grassroot workers like Accredited Social Health Activist (ASHA) and Anganwadi Worker (AWW). The Auxiliary Nurse Midwife (ANM) and Multi-Purpose Worker (MPW) were placed at subcentre and PHC level for execution of all the national programmes. The present control/elimination programmes of malaria, visceral leishmaniasis, and filariasis leverage on the existence of above-described delivery system. Thus, the human resources are common, although the program execution is totally compartmentalised.

### Pros and cons of vertical programmes

Some notable benefits include: (a) increased profile for high-priority vector-borne diseases in elimination mode viz malaria, lymphatic filariasis and kala azar; (b) it remains effective despite ill-equipped and weak public health systems; (c) an ability to cover neglected and inaccessible populations; (d) a positive side effect on health systems like strengthening surveillance systems and quality control of laboratories; (e) it allows for better monitoring and accountability via transparent governance arrangements; (f) it allows involvement of nongovernment organizations, civil society, philanthropic bodies, donors, partners, and other stakeholders; (g) enhancement of trust in health beneficiaries; and (h) it enables availability of resources including financial for other prevalent diseases [1,7].

The shortcomings of these vertical programmes are (a) the donor-driven programmes and global health initiatives (like GFTAM for malaria, nongovernment organizations like CARE and KalaCORE for visceral leishmaniasis) have created parallel systems of planning, functioning, information systems, monitoring, and evaluation frameworks influencing national policies adversely by diverting and deflecting the coordinated efforts of policy makers to strengthen health systems; (b) these deplete scarce human resources from mainstream health services especially for delivering time-bound activities like distribution of insecticide nets and mass chemotherapy for lymphatic filariasis; (c) they overburden the procurement and supply-chain management systems by unplanned overloading of the supply lines with diagnostics, drugs, or vector control products obtained from donor agencies. Usually, the existent systems are weak and not positioned to accept a huge influx and thus suffer temporary disruptions; (d) they fragment the health system by creating duplicate surveillance and other programmatic structures, thus making interprogrammatic coordination difficult; (e) they create negative spillover effects for the healthcare system by thwarting planned mechanisms for integration into mainstream health services, besides the potential of exclusion of nonparticipant/nonbeneficiary populations. For instance, provision of long-lasting nets to households with pregnant women and young children on a priority can make other residents feel left out; (f) the stakeholders with vested interests may obstruct reforms designed to integrate services, for example, an agency (funded by donors) that runs vector control operations like indoor residual spray would prefer to continue so as to justify its role and existence even if there is evidence of reduced effectiveness of the spray. Such an agency may also resist integration with other vector control operations for diseases although in the overall benefit of the national agenda. At times, stand-alone operations (as in the example above) may gather more visibility than integrated and merged programmes, and hence, these are sometimes preferred by private agencies; (g) they create differential pay and incentive structures (for example, cash incentives to community volunteer for diagnosis and complete treatment of kala azar patient) promoting neglect of routine work and may discourage staff in the general health system; (h) a top-down approach curbs initiatives by affected communities [1,7].

### Need for an integrated approach

**Fig 1:**
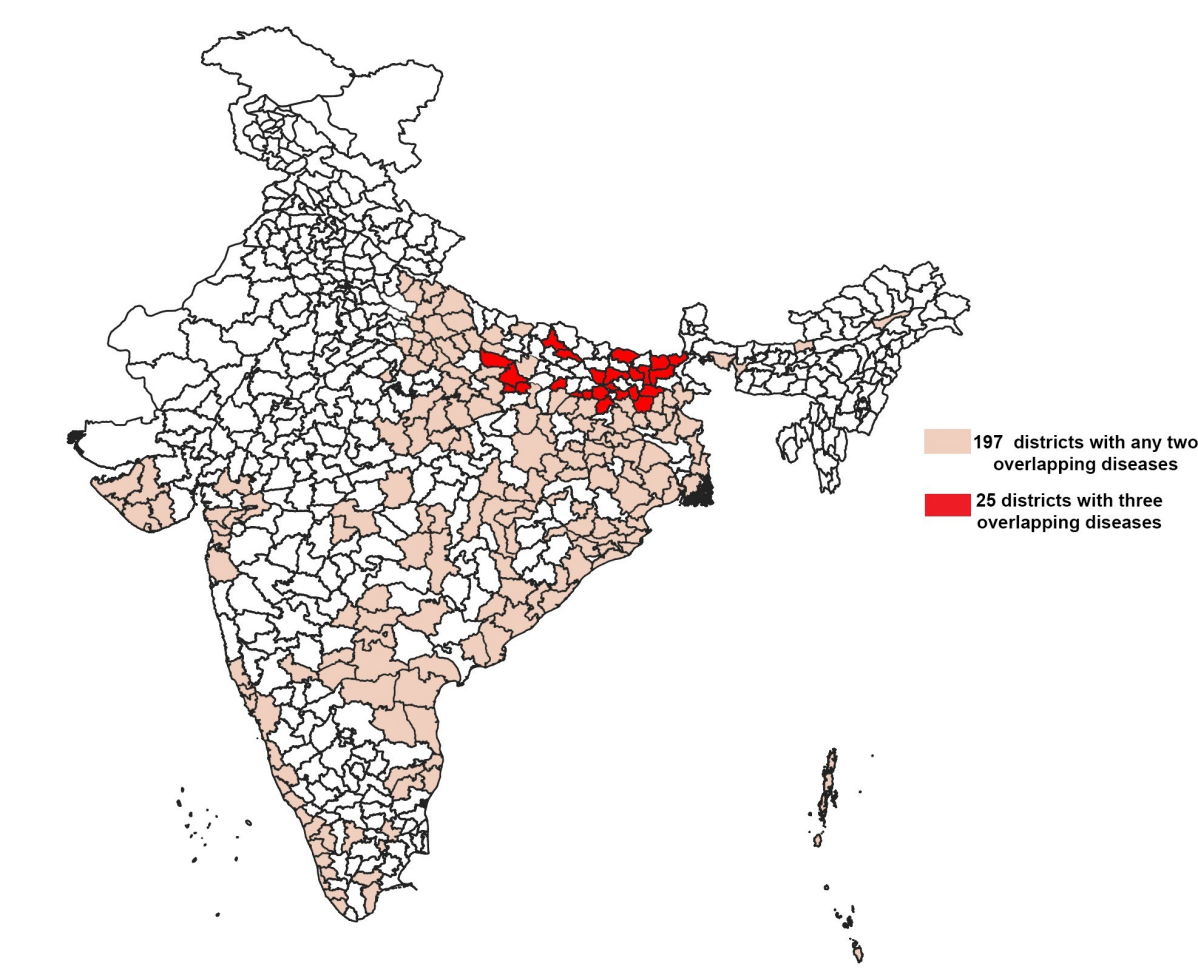
India map depicting the coendemic districts with malaria, kala azar, and lymphatic filariasis. The districts with any 2 diseases (light peach) and with 3 overlapping diseases (red) are marked on the map. The district map of India is provided by Data{Meet} Community Maps Project. It is made available under the Creative Commons Attribution 2.5 India (http://projects.datameet.org/maps/districts/).

In view of the above, it would be pragmatic for affected countries like India to devise a plan for a unified integrated control and elimination agenda. Integration can be defined here as amalgamation of the 3 disease-specific thrusts even beyond the concept of “integrated service delivery,” which focuses on providing health services in the same location by the same health workers [7]. It rather encompasses the entire spectrum of elimination programmes ranging from its implementation to governance and financing. Overlay of malaria and COVID-19 disease is a concern, and therefore, there are lessons for integration of other vector-borne diseases [8]. Integration can be viewed 2 ways—integration of the 3 vertical programmes—and in addition, integration with the general mainstream health services system. Leveraging vector-borne disease control programmes in a single programmatic structure to holistically address the 3 diseases offers a greater overall health benefit. Besides being parasitic diseases and in elimination mode, the 3 diseases share several components of disease epidemiology and control programmes. The overlapping coendemic areas and shared control strategies are an important impetus for integrating the programmes. Under the elimination agenda for these 3 diseases, the national programmes have rebooted and redesigned themselves from control to elimination in order to maximise coverage and deployment of control tools. On the other hand, the other 3 diseases, namely dengue, chikungunya and Japanese encephalitis, while being very important, display wide differences in their geographical areas, epidemiology, control measures, requirements of community engagement and therapies. In addition, these viral diseases lack definitive treatments, and we currently possess limited understanding of their pathology and progression. Hence we propose that for now malaria, visceral leishmaniasis and lymphatic filariasis be targeted for integration rather than all 6. Cross-disease integration of the 3 diseases, which are already rooted in the general healthcare system of the country, could be gainful. An integrated approach would be most suited for the following reasons and opportunities: (a) **Diminishing burden**: The malaria, kala azar, and lymphatic filariasis burden is shrinking and all are progressing towards elimination. With this other diseases like the current COVID19 pandemic would take priority for the policy makers. Joint elimination programmes of a common architecture would be far more cost-effective and may offer greater scope of scaling up [9]; (b) **Coendemic areas**: As shown in Fig 1, there are coendemic and congruent geographical areas where the 3 diseases overlap. As per data pertaining from 2016 to 2019, there are 25 districts with all 3 diseases and 197 districts with any of the 2 diseases (Fig 1). In addition to common geographies, several epidemiological features are common between the 3 diseases such as environmental, socioeconomic, demographic, cultural profile and health seeking behaviour of at-risk populations. This convergence makes it feasible and practical for the 3 control operations to function in one programmatic space. Joint surveillance (epidemiological and entomological surveillance), control and elimination activities in a comprehensive and holistic manner may yield greater success [9]; (c) **Augmentation of general healthcare services:** The proposed merger would significantly raise access to the healthcare systems by community as febrile illness episodes constitute a large proportion of the morbidity profile of the 3 infectious diseases. Being screened for common causes of fever under one umbrella as part of a broader healthcare objective would be an advantage. It will save repetitive work, resources, and can provide continual comprehensive services. Further, epidemiological information will travel in a more cohesive manner; (d**) Common challenges** exist like insecticide and drug resistance, need for better diagnostics, and role of private sector which poses as a gap in the surveillance system. Synergy with nonhealth sectors such as agriculture, housing, urban and rural planning and public works is currently missing but needed for well-rounded elimination programmes of all 3 elimination programmes.

### Intersecting components

**Fig 2:**
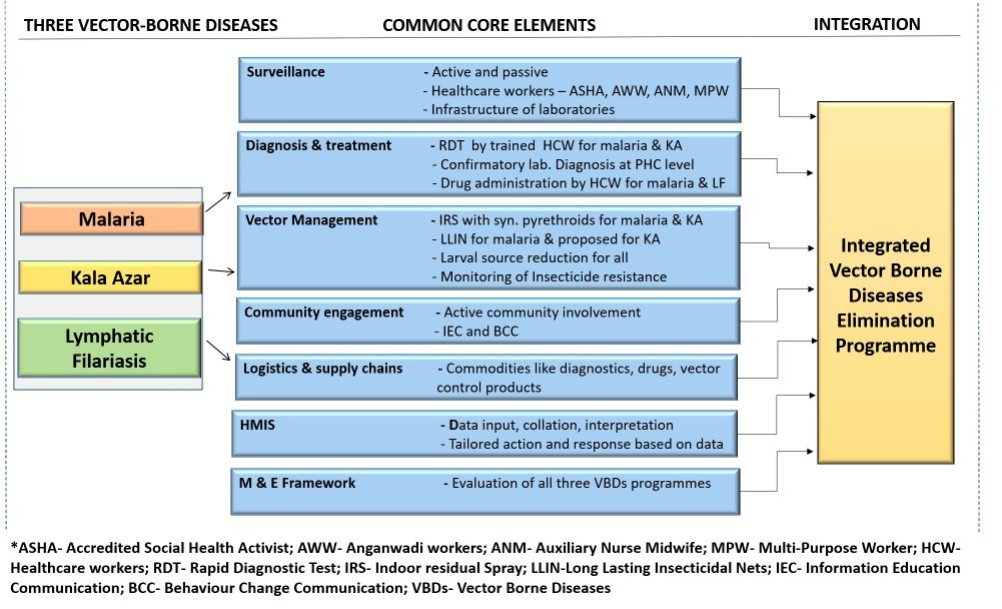
Integration of 3 vector-borne disease control programmes.

Given below are the strategies, their commonalities, and the feasibility of integration at an operational level [10–12]. The 3 vertical diseases control programmes can be transformed into one horizontal programme (Fig 2). Fig 2 depicts the vision for integration. There are core elements in each domain of programme implementation which are common and are likely candidates for integration.

1. **Surveillance:** Routine surveillance is through active (case detection by house-to-house visits) and passive surveillance (those reporting to the healthcare facilities). Different healthcare workers are engaged at various levels, namely health workers at primary level and above like ANM, MPW, medical officers and laboratory staff. Community volunteers like AWW and ASHAs also provide healthcare to the people at grassroots level. In addition, entomological surveillance and monitoring of insecticide resistance is commonly done for malaria and kala azar. Since the healthcare staff structures are same for the 3 diseases, surveillance mechanisms can be integrated and staff can be multitasked, thus conserving resources. As cases of malaria reduce and India aims at sustaining malaria control, near real-time surveillance and reporting will become more desirable. Epidemiological data from all partners and stakeholders including private sector, nongovernment organizations, philanthropic agencies, donors, military, railways, etc. will need to be collated centrally for comprehensive analysis and data-driven decision-making. One robust solution is the development of a digital dashboard platform where above data sets can be coalesced and made accessible for both research and public health experts. This envisioned dashboard can accommodate epidemiological data of all 3 parasitic diseases and can be visualised in real time for evidence-backed control actions. Efforts to make the surveillance near real time and holistic involving all stakeholders and using a digital platform have already been proposed [13]. Reporting formats can be modified and made comprehensive to include all the 3 diseases. When the data transfer would happen in near real time, digital dashboards would be very convenient and far more useful than paper-based reporting tools. The digital dashboards of COVID-19 have played a significant role in providing timely information to the scientific community and policy makers, and the concept can be replicated for malaria [14].
2. **Diagnosis and management**: Point-of-care tests are used for early detection of malaria and visceral leishmaniasis. The filarial test strip is used for lymphatic filariasis. However, for all 3 diseases, confirmatory laboratory diagnosis is done at a facility. The healthcare staff are trained for diagnostic tests in the field. Similarly, treatment is administered by healthcare workers in the community in case of malaria and to at-risk population of lymphatic filariasis in mass drug administration format. For kala azar, the treatment is facility-based since the current treatment is by single-day intravenous infusion. In addition, development of joint rapid diagnostic kits would indeed be very valuable. Multiplexed assays for the 3 infections, if available at point of care, or at the PHC would be very useful in addressing the “early detection” component of control programmes. Indeed, integration of research activities under the umbrella of disease elimination will be a visionary step wherein the operational gaps of the programme could be identified and solutions devised via prompt action-oriented research. In addition, asymptomatic carriers in all the 3 diseases are of concern both for the scientific community as well as for programme managers. Asymptomatic carriers may or may not progress to clinical disease, but these carriers do escape the surveillance systems as they do not report to the healthcare system. However, they do act as reservoirs of infection and likely fuel transmission of each disease. Therefore, it is important to develop and deploy diagnostic tools for asymptomatic carriers in the programmatic mode, especially when all 3 diseases are in elimination phase. Molecular tools suffer from the limitation of being resource intensive in terms of expertise and infrastructure required. However, there have been attempts at developing diagnostic tools as point-of-care tests or at least for PHC level tests. These inlcude LAMP for visceral leishmaniasis and TrueNat PCR tool for malaria. Detection methods for filariasis include night blood survey (which is inconvenient) and filarial test strip. For lymphatic filariasis, the triple drug in mass drug administration format (albendazole, DEC, and ivermectin) aims at achieving a rapid decline in microfilaria rate. However, it requires both high coverage and high compliance rate in the community. Hence, it is being gradually rolled out in the country in a phased manner. With a large number of endemic districts for lymphatic filariasis (256 districts), coverage will take considerable time. Hence, here again, the healthcare workers can be trained holistically for malaria, lymphatic filariasis and visceral leishmaniasis in context of diagnosis and treatment of the symptomatic and asymptomatic patients.
3. **Integrated vector management:** The routine vector control tools of IRS and long-lasting insecticide nets (LLINs) for malaria are being proposed for visceral leishmaniasis. Lymphatic filariasis vector control benefits from both of the above interventions. In addition, larval source management is common to all 3 programmes. Improved housing conditions have the potential to benefit all vector-borne diseases owing to reduction in favourable conditions for vector breeding. Susceptibility of vectors to the commonly used insecticides needs to be routinely monitored for all 3 diseases. Success of vector control programmes also depends on the acceptance and correct usage of the available tools by the community. Appropriate use of LLIN, acceptance of IRS, larval source management, and/or use of personal protective products are prerequisites for successful vector control. Therefore, joint vector management programmes, co-opted for the 3 diseases, x be far more advantageous than segmented individual programmes.
4. **Communities:** Encouraging community involvement for better healthcare is a vital and common element to the 3 programmes. Active community involvement via household visits, awareness camps, community and religious leaders, and drama and theatre are the usual strategies for malaria, lymphatic filariasis and visceral leishmaniasis. Since the latter 2 diseases have protracted clinical manifestations and are prone to delay in healthcare seeking by the affected, special efforts are made to raise the perception levels of the community. Morbidity management of lymphatic filariasis is the second pillar of elimination strategy (first being mass drug administration) and is deployed via healthcare workers. Thus, high levels of community engagement are essential for all 3 disease control programmes.
5. **Logistics and supply chains:** Diagnostics, drugs and vector control products utilize the same logistical and transport infrastructure available under the national programme. The vast geographical areas in India pose a challenge to timely provisions. Presently, the overstretched and fragile supply chains need to cater to demands of the 3 programmes. Once consolidated under one umbrella, the pressure on the supply networks may ease, and delivery of essentials may become more efficient.
6. **Monitoring and evaluation:** Performance of each of the 3 control programmes is assessed periodically although the basic indicators are broadly similar for each. An integrated framework can be envisioned that will accommodate assessment of all 3 simultaneously. Since the surveillance strategies and intervention components will be shared, the monitoring and evaluation can also be assimilated. Integration of this component will be resource efficient and will provide government updates of the 3 eliminable diseases in a broad overarching manner.
7. **Health Management Information System:** The existing information networks for the 3 diseases comprise of data aggregation from peripheral level (PHC) to centre (block, district, and state level). The data collection and collation is paper-based and monthly. World Health Orgnanization recommends countries to switch to near real-time data transmission for timely evidence-based decisions. This holds true for all 3 diseases in elimination mode as timely information and data-backed interventions are crucial for the success of the programmes. Information systems, though fragmented and outdated at present, can be made cohesive and data collection can be integrated. Digital platforms would make the convergence of data generated in the 3 disease systems feasible and available for analysis, interpretation and action.

## Conclusions

As India marches towards the goal of elimination of kala azar (2021), lymphatic filariasis (2021), and malaria (2030), there is a need to reorient and reposition our programmatic structures. Their governance should aim to assimilate the 3 vertical, stand-alone programmes into a single horizontal, and integrated programme. Integration of data from all sources including the private sector and other government sectors is needed for complete information on disease burden and for data-driven decisions [13]. It is an opportune time for India to make this transition from segmented programmes to a consolidated single programme for vector-borne diseases in the elimination mode. The 3 diseases control aims have been historically segregated and have been vertical in their approaches. Cross-disease unification of the 3 diseases has its own challenges and would require major shifts and repositioning of an entire gamut of healthcare systems ranging from policy to structures. However, it is very much realizable and should be attempted as integration of malaria, lymphatic filariasis and visceral leishmaniasis control programmes would be a judicious and progressive step in the right direction.
