# Correction: India can consider integration of three eliminable disease control programmes on malaria, lymphatic filariasis, and visceral leishmaniasis

**DOI:** 10.1371/journal.ppat.1010984

**Published:** 2022-11-21

**Authors:** Manju Rahi, Rini Chaturvedi, Payal Das, Amit Sharma

The ORCID iD for the corresponding author, Amit Sharma, is incorrect. The correct ORCID iD is: 0000-0002-3305-0034 (https://orcid.org/0000-0002-3305-0034).
